# Synthesis and Characterization of Nanocomposite Hydrogels Based on Poly(Sodium 4-Styrene Sulfonate) under Very-High Concentration Regimen of Clays (Bentonite and Kaolinite)

**DOI:** 10.3390/gels10060405

**Published:** 2024-06-18

**Authors:** Tulio A. Lerma, Enrique M. Combatt, Manuel Palencia

**Affiliations:** 1 Research Group in Science with Technological Applications (GI-CAT), Department of Chemistry, Faculty of Natural and Exact Sciences, Universidad del Valle, Cali 760042, Colombia; 2 Mindtech Research Group (Mindtech-RG), Mindtech s.a.s., Monteria 230003, Colombia; 3 Department of Agricultural Engineering and Rural Development, Universidad de Córdoba, Monteria 230002, Colombia; ecombatt@fca.edu.co

**Keywords:** soil polymer conditioner, organoclay, hybrid composite, geomimetic

## Abstract

The aim of this work was to synthesize and study the functional properties of polymer-clay nanocomposite (PCNCs) based on poly(sodium 4-styrene sulfonate) (NaPSS) and two types of clay in the dispersed phase: bentonite and kaolinite, in order to advance in the development of new geomimetic materials for agricultural and environmental applications. In this study, the effect of adding high concentrations of clay (10–20 wt. %) on the structural and functional properties of a polymer–clay nanocomposite was evaluated. The characterization by infrared spectroscopy made it possible to show that the PCNCs had a hybrid nature structure through the identification of typical vibration bands of the clay matrix and NaPSS. In addition, scanning electron microscopy allowed us to verify its hybrid composition and an amorphous particle-like morphology. The thermal characterization showed degradation temperatures higher than ~300 °C with Tg values higher than 100 °C and variables depending on the clay contents. In addition, the PCNCs showed a high water-retention capacity (>2900%) and cation exchange capacity (>112 meq/100 g). Finally, the results demonstrated the ability of geomimetic conditioners to mimic the structure and functional properties of soils, suggesting their potential application in improving soil quality for plant growth.

## 1. Introduction

Polymer–clay nanocomposites (PCNCs) are hybrid organo-inorganic systems formed by dispersion of one clay, into relatively small quantities, in a polymer-nature continuous phase. In these materials, the dispersed phase has a marked effect on the mechanical, barrier and thermal properties, which usually increase, reach a maximum value, and subsequently experience a decrease as clay content is increased. The maximum value is generally identified between 3 and 5 wt. %, therefore, studies for the development of PCNCs at higher clay concentrations are very scarce (i.e., 5–10 wt. %), but also, these are practically non-existent for very high clay concentration regimens (upper to 10 wt. %). The above is mainly due to the rise of structural materials for applications where mechanical, barrier and thermal properties are desired; however, within the framework of functional polymers, the areas of application, processability and properties of interest go beyond those properties previously mentioned. Some examples are hydrogels, ionic exchangers, membranes, sensors, among others [1,2].

On the other hand, soil plays a vital role in supporting terrestrial ecosystems and sustaining life on Earth. According to the Food and Agriculture Organization of the United Nations (FAO), it is estimated that by 2050, agriculture will have to double the production of food, livestock fodder and biofuels; in addition, to transform the way food is processed and consumed to meet the needs of approximately ten billion people in the world [3]. However, to date, excessive and indiscriminate practice of anthropogenic activities on soils, including industrialization and agriculture, has led to the alteration of their properties and their undeniable degradation, which currently poses important environmental challenges [4]. Furthermore, in places where the degree of soil degradation is very high, the expansion of the agricultural frontier becomes relevant, generating deforestation, soil erosion, nutrient depletion, and alteration of environmental biodiversity [5].

In recent decades, practices to improve physicochemical properties, increase soil fertility and promote healthy plant growth have been developed and promoted, which are based on crop rotation practices, the use of crops of coverage, the application of no-till agriculture, precision agriculture, among others [6]. In fact, practices based on the use of agrochemicals are widely used to increase crop yields; however, excessive, and indiscriminate use generates harmful effects on the health of soils and surrounding water bodies [7]. Consequently, soil conditioners have acquired relevance through being used as natural or synthetic materials, which modulate different characteristics of the soil, such as its structure, texture, water retention capacity, pH, and nutrient content. Among the best-known conditioners, a wide variety of materials stand out, such as compost, manure, peat, clays, polymers, and various types of organic and inorganic materials [8].

Particularly, the use of PCNCs has gained attention as a promising approach to address problems related to the restoration of degraded soils [9,10]. Furthermore, the progress in the development of geomimetic soil conditioners (materials whose design and function are inspired by natural systems of geological origin, e.g., the soil, and allow improvement in the physicochemical properties of degraded soils or soils with low agricultural suitability) has allowed progress in the construction of materials based on PCNCs capable of imitating the structures, biological/physicochemical properties, or functions of soils [11,12,13]. Thus, among the countless combinations that have not yet been explored, the synergistic interaction between NaPSS and clays has attracted significant attention towards the development of advanced materials [14,15].

Particularly, in soil science and agriculture Poly(sodium 4-styrene sulfonate) (NaPSS) has been gaining great interest for its ability to improve soil structure, water retention, and nutrient availability [16]. In addition, NaPSS is widely used as a model polyelectrolyte (polymers containing ionic and/or ionizable functional groups) system [17], as a flocculating agent to eliminate heavy metals and other contaminants in water treatment processes [18,19], as raw material for manufacturing of ion exchange membranes [20], personal care products and medicines [21]. The NaPSS is characterized by having an alkyl main chain with aryl sulfonate groups. Thus, sulfonate groups give it exceptional properties including high solubility, biocompatibility, and ion exchange capacity, among others. By virtue of these groups, NaPSS exhibits ion exchange properties allowing it to interact with soil particles and humified organic matter, promoting flocculation of colloidal soil particles and subsequent stabilization of aggregates, and finally influencing soil properties and ecosystemic functions. Therefore, the use of NaPSS in soils promises to be an alternative to mitigate soil erosion, improve fertility and promote plant growth [22].

On the other hand, clays are particles of aluminosilicates minerals that are very abundant in nature with a diameter less than 0.002 mm. Clays encompass a diverse group of natural minerals with layered structures, including montmorillonite, kaolinite, smectite, among others. The unique arrangement of layers provides clays with exceptional properties, such as high surface area, swelling capacity and ion exchange capacity [23,24]. Specifically, bentonite and kaolinite are two types of clays with significant differences in their chemical composition, crystal structure, and physicochemical properties, making them useful in the petroleum and gasification industry for the purposes of ceramic manufacturing and wastewater treatment [25,26]. Thus, bentonite is a clay rich in montmorillonite minerals, with cation exchange capacity due to its 2:1 laminar structure, composed of an octahedral layer of alumina between two tetrahedral layers of silica with the presence of intercalated ions [27]. Additionally, it has a high capacity to absorb water, making it valuable in oil and gas drilling applications. In contrast, kaolinite, known for its whiteness and fineness, has a 1:1 lamellar structure, a tetrahedral layer of silica and another octahedral layer of alumina; however, it has low adsorbent properties due to its low specific surface area and minimal isomorphic substitution, which gives it high molecular stability, low shrinkage, plasticity, swelling and cohesion, making it valuable in the manufacture of ceramics, paper, and ceramic products [28].

Based on the above, the combination of NaPSS with clay emerges as an opportunity to create PCNCs with synergistic properties that exceed those of the individual components, providing improvements in their mechanical resistance, thermal stability, and functional properties; but also, these can be used in the improvement and restoration of degraded soils. Thus, the objective of this work was to synthesize and study the functional properties of clay–polymer composites based on NaPSS and two types of clay in the dispersed phase: bentonite and kaolinite, to advance the development of new geomimetic materials for agricultural and environmental applications. For this, clays were modified with organosilanes for the incorporation of vinyl groups in its structure, which act as anchor points for the growth of NaPSS polymer chains. Thus, here it is proposed that PCNCs can be used to imitate the clay-humin-humic acid structures present in soil particles and improve their cationic exchange properties, water retention and aggregate formation.

## 2. Results and Discussion

### 2.1. Organoclays Synthesis

**Figure 1:**
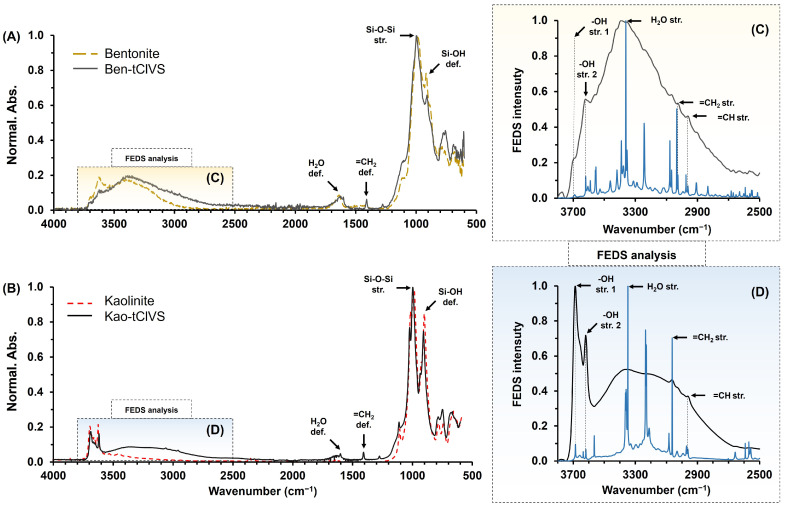
IR-ATR spectra of bentonite (dashed line) and bentonite–tClVS (solid line) (**A**) and kaolinite (dashed line) and kaolinite–tClVS (solid line) (**B**). IR-ATR-FEDS spectra of bentonite–tClVS (**C**) and kaolinite–tClVS (**D**) between 2500 and 3800 cm^−1^.

Figure 1A,B show IR-ATR spectra of unmodified bentonite and kaolinite (dashed lines) and modified with tClVS (solid line), respectively. Thus, in the analysis of the region below 2000 cm^−1^ in all of the cases spectra, at ~988 cm^−1^ and ~908 cm^−1^ the presence of two characteristic bands of clay minerals can be seen; these signals were associated with the tension vibration of Si-O-Si groups and deformation vibration of Si-OH groups, respectively. Likewise, at ~1632 cm^−1^ the deformation vibration signal of the interlaminar water molecules contained by the clay and organoclays was identified, this being of lower intensity for the kaolinite spectra [29]. Finally, only in the spectra of clays modified with tClVS (solid line), a weak signal was identified at ~1404 cm^−1^, linked to the in-plane deformation vibrations of the vinyl group, =CH_2_, anchored on the surface of the clays. Now, for the analysis of the spectral region between 2500 and 3800 cm^−1^, FEDS analysis was used for adequate identification and assignment of signals in areas of high overlap [30]. The IR-ATR-FEDS spectra of bentonite–tClVS and kaolinite–tClVS in the selected analysis region are shown in Figure 1C and Figure 1D, respectively. In general, in both spectra the presence of typical signals of aluminosilicates were observed, which differ in intensity because of the structural differences between bentonite and kaolinite, which alter their ability to interact and absorb water molecules [31]. At ~3682 and ~3621 cm^−1^, the tension vibration signals of hydroxyls from silanol groups and clays were identified, respectively. Likewise, at ~3436 cm^−1^, a signal associated with tension vibrations of absorbed water molecules was observed for both spectra. In the spectral region below 3100 cm^−1^, characteristic signals of organic vinyl functional groups incorporated into bentonite and kaolinite were observed. Thus, the tension vibration signal from the =CH_2_ group was observed at ~3060 cm^−1^, whereas a symmetric and asymmetric tension vibration signal from the =CH group was identified at ~2960 cm^−1^.

Based on the above, the identification of typical spectral bands of the C-H bonds belonging to the vinyl group (organic nature fragment) in the spectra of the organoclays allowed the verification of their adequate modification. Thus, the signals are consistent with the procedure for modification of clays with tClVS to obtain organoclays with the ability to react by free radical polymerization. Note that tClVS is characterized by having chlorine atoms in its structure in Si-Cl bonds, which can be anchored covalently by nucleophilic substitution reactions on clay surface; but also, tClVS has a vinyl group in its structure, which can intervene in free radical polymerization reactions. Thus, tClVS acts as a covalent bonding molecule or a coupling agent between the inorganic–organic fractions of composites and allows the obtaining of materials with greater stability in contrast to materials obtained through non-covalent interactions [32,33].

**Figure 2:**
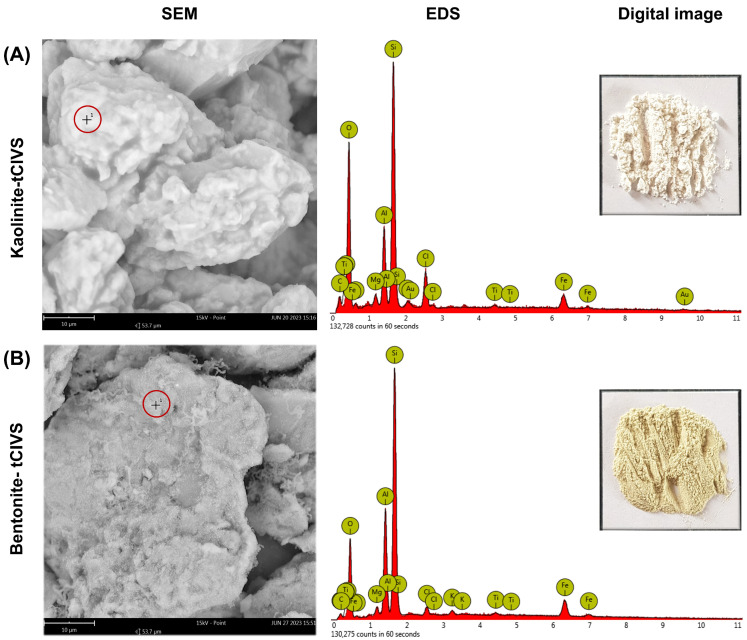
SEM images (left imagen), EDS spectrum for the determination of elemental composition (bottom right image, EDS analysis zone corresponds to the cross enclosed in the red circle in the SEM image) and digital images (top right image) of bentonite and kaolinite modified with tClVS: kaolinite–tClVS (**A**) and bentonite–tClVS (**B**).

On the other hand, Figure 2 shows the results of morphological analysis by SEM (image left), the EDS spectrum for determination of elemental composition and a digital image (top right image) for both kaolinite–tClVS and bentonite–tClVS. The EDS analysis zone corresponds to the cross enclosed in the red circle in the SEM image. Thus, at the macroscopic level, from the digital images in Figure 2, it can be seen that tClVS-modified clays did not show significant changes in their appearance after modification. Only the bentonite–tClVS organoclay showed a slight lightening in its color after synthesis; coloration caused by the presence of iron ions in its structure and their loss during washing processes [34]. At the microscopic level in the SEM images, a wide distribution in sizes and the absence of a defined shape were observed in kaolinite–tClVS and bentonite–tClVS particles (Figure 2A and Figure 2B, respectively). Regarding the elemental characterization by EDS of the tClVS-based organoclays, the results are shown in Table 1. In general, it was observed that the kaolinite–tClVS and bentonite–tClVS organoclays presented a higher content of Si and O in contrast to the elements Al and Fe, all of them being typical constituents of aluminosilicates [35]. Furthermore, it was possible to determine by EDS analysis the presence of characteristic elements of tClVS, i.e., C and Cl, in the structure of the organoclays. Thus, C and Cl contents for kaolinite–tClVS were 8.65% and 5.74%, respectively; for its part, in the same order, the values for bentonite–tClVS were 7.72% and 2.46%.

Finally, Table 1 shows the results of particle size and Z potential which were measured through DLS for both bentonite–tClVS and kaolinite–tClVS as their precursors. Initially, it was observed that bentonite and kaolinite had particle sizes of 268 ± 16 nm and 555 ± 20 nm, respectively. However, after modification with tClVS, it was observed that the obtained organoclays showed an increase in their size. Thus, bentonite–tClVS and kaolinite–tClVS presented particle sizes of 1144 ± 333 nm and 776 ± 34 nm, respectively. This variation and the increase in particle size are due to changes in the surface energies of clay particles caused by the incorporation of organosilane tClVS [36]. Covalent insertion of tClVS modifies the hydrophilic groups on the surface of the clay particles, decreasing their affinity for aqueous media and promoting their agglomeration [37,38]. On the other hand, the zeta potentials obtained at natural pH for bentonite–tClVS (−33.3 ± 4.7 mV) and kaolinite–tClVS (−16.6 ± 2.3 mV) showed a decrease in the measured values with respect to the unmodified clays: bentonite (−38.3 ± 0.2 mV) and kaolinite (−38.8 ± 1.3 mV). The decrease in the potentials is attributed to changes caused at the surface level of the clay minerals because of incorporating tClVS, which decreases the density of polar groups and increases the nonpolar fraction after the incorporation of vinyl groups [39,40]. Thus, the determination of the typical vibration bands of the vinyl organic groups through IR-ATR-FEDS, the identification of the C and Cl elements in the structure of the organoclays through EDS, and the identification of variations in the particle size and Z potential by DLS, allowed for verification of the adequate synthesis of organoclays from tClVS with bentonite or kaolinite.

### 2.2. Geomimetic Soil Conditioners Synthesis

After being obtained, bentonite–tClVS and kaolinite–tClVS were used together with NaSS for the synthesis of geomimetic conditioners. For this, the preparation of geomimetic conditioners was carried out through the formation of hybrid composites using the in situ polymerization technique, that is, the NaSS was polymerized via free radicals in the presence of kaolinite–tClVS or bentonite–tClVS; thus, the anchoring of NaPSS chains to the surface of the clays and their adequate dispersion in the material was performed [41,42]. In this work, clay–NaPSS hybrid composites were synthesized with clay proportions of 10.0 and 20.0% (i.e., under very high concentration regimen). The composition and name of the geomimetic conditioners are given in Table 2.

**Figure 3:**
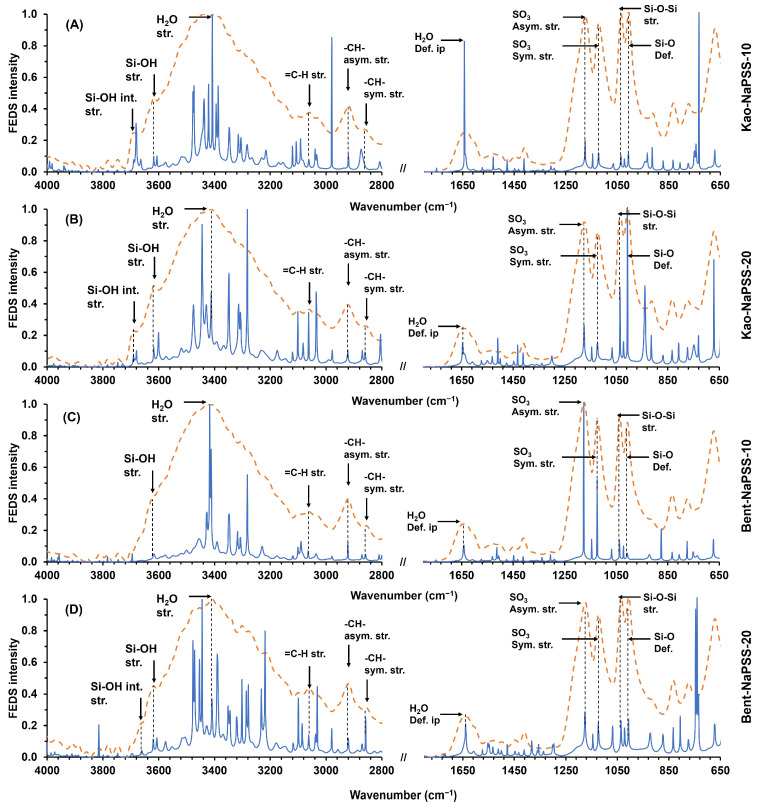
IR-ATR-FEDS spectra of PCNCs: Kao-NaPSS-10 (**A**), Kao-NaPSS-20 (**B**), Bent-NaPSS-10 (**C**) and Bent-NaPSS-20 (**D**). Dashed line IR-ATR spectra and solid line FEDS spectra. (being Kao = kaolinite, Bent = bentonite, and NaPSS = sodium poly(styrene sulfonate), where clay percentages are given by 10 and 20 in the end of each notation).

Figure 3 shows the IR-ATR-FEDS spectra of PCNCs Kao-NaPSS-10 (A), Kao-NaPSS-20 (B), Bent-NaPSS-10 (C), and Bent-NaPSS-20 (D) (being Kao = kaolinite, Bent = bentonite, and NaPSS = sodium poly(styrene sulfonate), where clay percentages are given by 10 and 20 in the end of each notation). In general, typical signals of the organic group’s constituents of the NaPSS matrix were observed in all spectra; as well as characteristic signals of the bentonite–tClVS and kaolinite–tClVS. In both cases, it was possible to identify at ~3675 cm^−1^ and ~3610 cm^−1^ the stretching vibrations of silanol groups and interlaminar hydroxyl groups constituting the clays. In addition, two high-intensity signals related to tension vibrations of Si-O-Si groups (~1036 cm^−1^) and deformation vibration of Si-OH groups (~988 cm^−1^) were observed. All the above signals are characteristics of clay fraction in the composites [43].

On the other hand, in all the IR-ATR-FEDS spectra of the all synthesized PCNCs shown in Figure 3, it was possible to identify at ~3050 cm^−1^ the stretch vibration of the C-H bond from NaPSS aromatic ring. In addition, signals of asymmetric and symmetric stretch vibrations, at ~2940 cm^−1^ and ~2860 cm^−1^, respectively, related to C-H bonds of methyl and methylene groups were observed. Likewise, symmetric and asymmetric stretch vibrations of sulfonate groups were observed at ~1170 cm^−1^ and ~1116 cm^−1^, respectively [44]. From the above, it is possible to corroborate the inorganic-organic hybrid constitution of the synthesized materials. Finally, stress and strain vibration signals of water molecules constituting the clay fraction and are occluded in NaPSS matrix were observed at ~3430 cm^−1^ and ~1650 cm^−1^.

**Figure 4:**
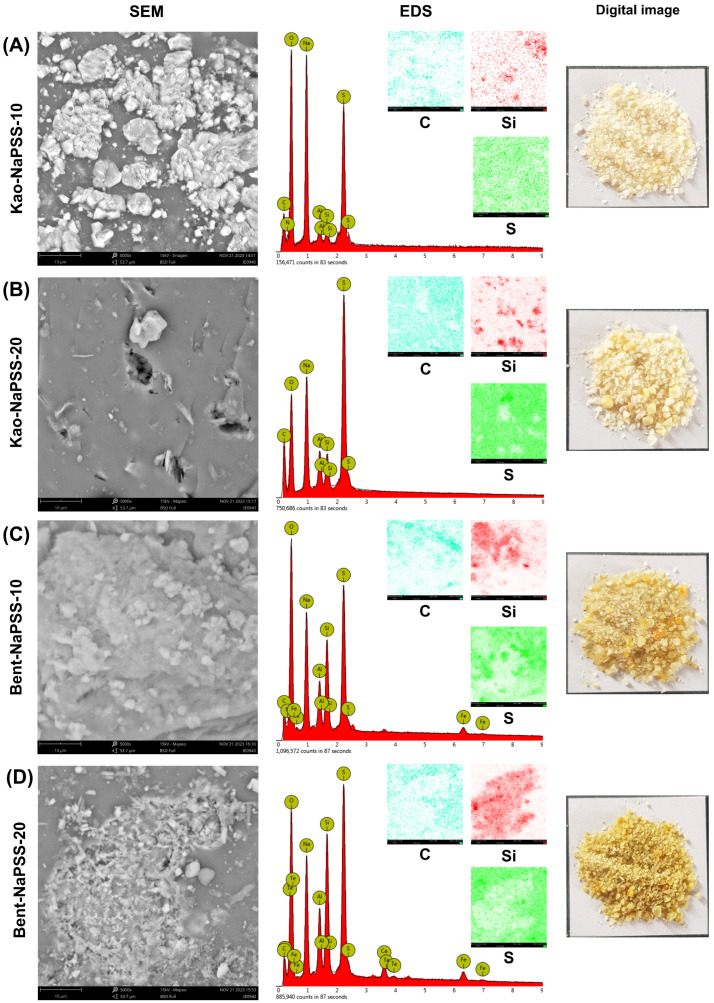
SEM images (left image), EDS spectrum for the determination of elemental composition (bottom center image, EDS analysis was carried out in mapping mode of the corresponding SEM image), results of EDS mapping (upper center images, each color represents an element, C (carbon), S (sulfur) and Si (silicon)) and digital images (right image) of PCNCs: Kao-NaPSS-10 (**A**), Kao-NaPSS-20 (**B**), Bent-NaPSS-10 (**C**) and Bent-NaPSS-20 (**D**).

Continuing with morphological and elemental characterization of PCNCs, Figure 4 shows images obtained by digital photography (right image) and SEM (left image), EDS spectrum (bottom center image) and results of EDS mapping (upper center images) obtained from PCNCs: Kao-NaPSS-10 (A), Kao-NaPSS-20 (B), Bent-NaPSS-10 (C) and Bent-NaPSS-20 (D). In general, it was observed that materials obtained at a macroscopic level presented a white-cream hue for those based on kaolinite–NaPSS and a more intense white-yellow hue for bentonite–NaPSS composites. In both cases, the materials with clay contents of 20.0% *w*/*w* were those that presented the highest intensity. Likewise, irregular particulates with a size less than 2.0 mm were observed for all materials. Now, at the microscopic level, the results of SEM analysis showed that PCNCs had fractions of bentonite and kaolinite in their structure. Furthermore, when distribution profiles of Si were analyzed, it was observed that it was distributed throughout the polymer matrix; however, some areas of greater intensity were characterized by higher Si contents, evidencing shortcomings related to the clay dispersion process in the synthesis of composites [45].

Finally, results of compositional analysis of PCNCs are shown in Table 2. The results obtained show that the C, S, Na and O contents represent the majority in contrast to the Si and Al contents. Particularly, NaPSS is constituted by C, H, S, O and Na, and because of it is main component of PCNCs, a high compositional similarity between these materials and pure NaPSS is expected. Likewise, the presence of non-constituent elements of NaPSS provides evidence that the inclusion of bentonite and kaolinite into the polymer matrix has occurred.

### 2.3. Geomimetic Soil Conditioners Thermal Characterization

**Figure 5:**
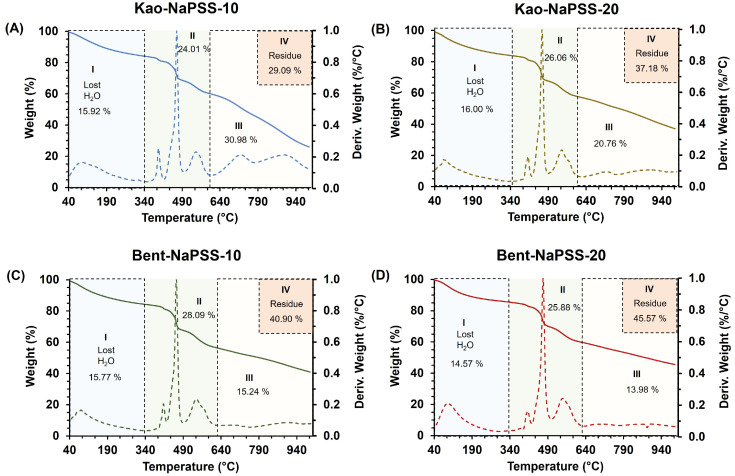
TGA results (solid line) and derivative thermogravimetric curve (dashed line, mass/temperature derivation) of PCNCs: Kao-NaPSS-10 (**A**), Kao-NaPSS-20 (**B**), Bent-NaPSS-10 (**C**) and Bent-NaPSS-20 (**D**).

Figure 5 shows the results of thermal characterization by TGA (solid line) and derivative thermogravimetric curve (dashed line, mass/temperature derivation) of synthetized PCNCs: Kao-NaPSS-10 (A), Kao-NaPSS-20 (B), Bent-NaPSS-10 (C) and Bent-NaPSS-20 (D). The results showed that the hybrid materials had four stages of mass loss related to water loss and structural degradation. Thus, kaolinite-based composites, namely Kao-NaPSS-10 and Kao-NaPSS-20 (see Figure 5A and Figure 5B, respectively), showed an initial stage of mass loss with a loss of ~16%. This loss was associated with the elimination of water molecules occluded in the polymer matrix and absorbed in clay minerals. Next, a second stage of mass loss was observed, that begins around ~340 °C and extends to ~600 °C, presenting losses of 24.06% and 26.06% for Kao-NaPSS-10 and Kao-NaPSS-20, respectively. Likewise, a third stage of mass loss was observed, in temperature ranges from ~600 °C to ~1000 °C, of 30.98% and 20.76%, for Kao-NaPSS-10 and Kao-NaPSS-20, respectively. According to previous studies, it has been reported that the thermal degradation of NaPSS occurs in two stages: the first occurs above 200 °C to below 600 °C and is characterized by the breaking of the C-S bonds of polymer chains, generating the formation of radicals and the release of sulfur dioxide. Next, a second stage of degradation occurs above 600 °C and extends up to 1000 °C; this is attributed to the degradation of the carbonated polymer chain of styrene, generating the release of gases rich in styrene, methyl styrene, toluene, and benzene [46]. Thus, according to the behavior observed for Kao-NaPSS-10 and Kao-NaPSS-20, degradation profiles are similar to those presented by NaPSS and those that overlap with the typical degradation processes of clays [28]. Finally, above 1000 °C, a residue of 29.09% and 37.18% was observed for Kao-NaPSS-10 and Kao-NaPSS-20, respectively, which is attributed to carbonaceous material and calcined kaolinite residues [47].

On the other hand, thermal characterizations of bentonite-based PCNCs, Bent-NaPSS-10 and Bent-NaPSS-20, Figure 5C and Figure 5D, respectively, are summarized in Table 3. In general, the degradation profiles of composites obtained from bentonite showed a behavior like that observed in those obtained from kaolinite. These presented three regions of mass loss and generation of a residue above 1000 °C. However, it was observed that the amount of mass lost in the third stage was lower for these samples. In addition, a greater quantity of residue was obtained, with values of 40.90% and 45.57% for Bent-NaPSS-10 and Bent-NaPSS-20, respectively. Thus, since the thermal degradation processes of the NaPSS matrix and the bentonite clay fraction are overlaid; these variations are associated with the structural differences between bentonite and kaolinite that modulate their water loss and thermal dehydroxylation processes [48,49].

**Figure 6:**
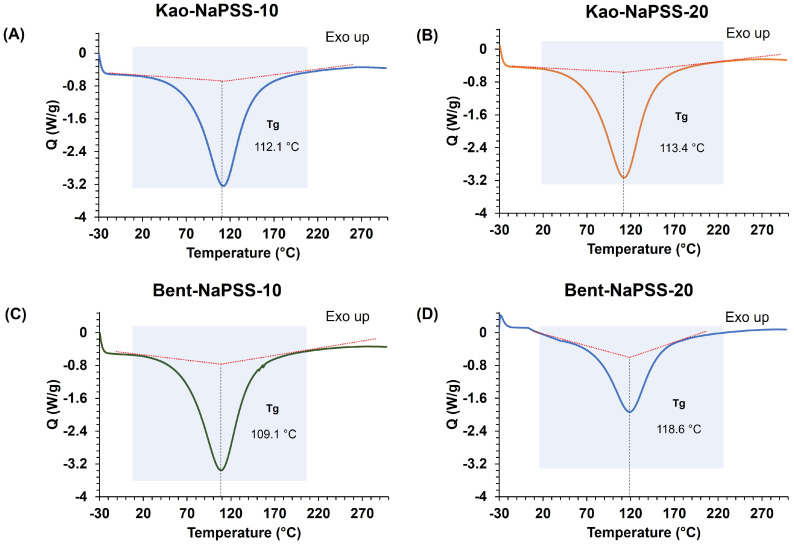
DSC results of PCNCs: Kao-NaPSS-10 (**A**), Kao-NaPSS-20 (**B**), Bent-NaPSS-10 (**C**) and Bent-NaPSS-20 (**D**). Analysis conditions: inert nitrogen atmosphere in a temperature range from room temperature to 300 °C and a heating ramp of 15 °C/min.

Finally, the results of DSC characterization of the PNCNs based on bentonite–NaPSS and kaolinite–NaPSS are shown in Figure 6. In general, an effect of the clay/NaPSS composition on the glass transition temperature (Tg) of synthesized composites was observed. Thus, for Kao-NaPSS-10 and Kao-NaPSS-20 composites, the Tg values were 112.1 °C and 113.4 °C, respectively (see Figure 6A and Figure 6B, respectively). Likewise, an increase in Tg was observed between the bentonite-based composites, recording Tg values of 109.1 °C and 118.6 °C for Bent-NaPSS-10 and Bent-NaPSS-20, respectively (see Figure 6C and Figure 6D, respectively). The increase in the Tg of composites can be explained by the insertion of bentonite and kaolinite particles into the NaPSS matrix. It has been reported that NaPSS without reinforcing materials in its structure presented Tg values of 69.5 °C [50]. Thus, the application of an in situ polymerization technique of NaSS and the insertion of organoclay with surface vinyl groups allowed for an increase in the degree of cross-linking and interaction among NaPSS polymer chains in the composite. This generated a decrease in the mobility of polymer chains and an increase in the temperature required to generate the thermal transition.

### 2.4. Geomimetic Soil Conditioners Functional Characterization

**Figure 7:**
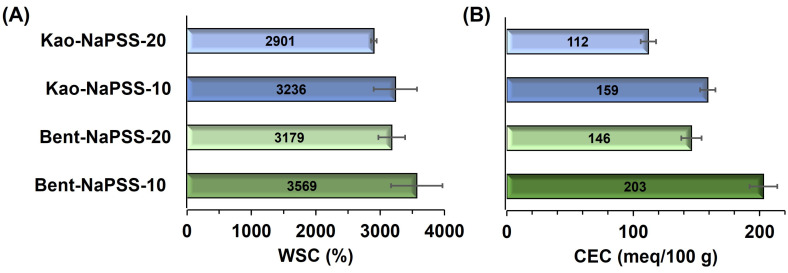
Water sorption capacity (WSC) (**A**) and cation exchange capacity (CEC) (**B**) of PCNCs: Kao-NaPSS-10, Kao-NaPSS-20, Bent-NaPSS-10 and Bent-NaPSS-20. The value in the center of the boxes and the error bars corresponds to the mean value and standard deviation of the measurements, respectively.

The results of functional characterization of WSC and CEC of PCNCs are shown in Figure 7A and Figure 7B, respectively. The values of WSC from Kao-NaPSS-10, Kao-NaPSS-20, Bent-NaPSS-10, and Bent-NaPSS-20 were 3236 ± 337%, 2901 ± 43%, 3569 ± 400%, and 3179 ± 207%, respectively. When comparing the values obtained with those reported in Table 1 for the kaolinite–tClVS and bentonite–tClVS, the high hydrophilicity that NaPSS gives to the composites is evident, which allows it to retain more than 29 times its own weight. Thus, NaPSS contains sulfonate groups in its structure that allow it to interact and absorb large amounts of water [51]. Finally, values of CEC from PCNCs, shown in Figure 7B, were higher when the clay contents were lower (10%), being for each case the following: Kao-NaPSS-10 (159 ± 6 meq/100 g), Kao-NaPSS-20 (112 ± 6 meq/100 g), Bent-NaPSS-10 (203 ± 11 meq/100 g) and Bent-NaPSS-20 (146 ± 8 meq/100 g). Thus, when comparing the values obtained by clay–NaPSS composites with the values reported in Table 1 for the organoclays and their precursors, it can be observed that the insertion of NaPSS into materials produced a significant increase in the CEC. Therefore, the presence of sulfonate groups in the NaPSS polymer chains, with the capacity for ionization and development of a permanent negative charge, gives the composites the ability to interact electrostatically with cations and generate ion exchange processes [50]. Therefore, previous results demonstrate the potential of PCNCs based on bentonite–NaPSS and kaolinite–NaPSS to mimic cation exchange processes as well as the water storage capacity of soils, which can be understood as fundamental processes for the proper growth and development of plants [52].

## 3. Conclusions

PCNCs are synthetic materials based on clay and functional polymers which are a promissory alternative for the making of geomimetic soil conditioners. These are synthetic geomaterials with hydrogel properties and CEC, they can be easily synthesized from NaPSS and surface-modified clays such as bentonite and kaolinite. The functionalization of clays is performed by direct reaction of clay and organosilanes compounds with vinyl groups in their structure. Thus, the presence of vinyl groups on the clay surface allows the anchoring of polymer chains and the formation of materials with greater stability due to the formation of covalent bonds. Characterization by IR-ATR spectroscopy and morphological analysis revealed the hybrid nature of the materials and an adequate dispersion of clays into the polymer matrix. Furthermore, the results of thermal characterization showed the influence of the clay/NaPSS ratio on the thermal properties of PCNCs, evidencing an increase in the Tg with the increase in clay content. Finally, the functional evaluation of PCNCs demonstrated the ability of geomimetic conditioners to retain high amounts of water and carry out cation exchange processes, which suggests their potential application in improving soil quality for plant growth.

## 4. Materials and Methods

### 4.1. Reagents and Materials

Bentonite (BentoCol S.A.S, Bugalangrande, Colombia) and kaolinite (Caolines Superior Boyacá S.A.S, Tunja, Colombia) and trichlorovinylsilane (H_2_C=CHSiCl_3_, tClVS, 97 wt. %, Aldrich, Milwaukee, WI, USA) were used as precursors of organoclays. Sodium 4-styrenesulfonate (NaSS, >90 wt. %, Aldrich, Milwaukee, WI, USA) and ammonium persulfate ((NH_4_)_2_S_2_O_8_, wt. ≥98.0%, Merck, Darmstadt, Germany) were used for the synthesis of geomimetic soil conditioners. Ammonium acetate (CH_3_COONH_4_, ≥98%, Merck, Darmstadt, Germany), sodium chloride (NaCl, ≥99 wt. %, Merck, Darmstadt, Germany), aqueous formaldehyde (HCHO, 37 wt. %, Merck, Darmstadt, Germany), sodium hydroxide (NaOH, ≥98 wt. %, Merck, Darmstadt, Germany), and potassium phthalate monobasic (HOOCC_6_H_4_COOK, 99.95–100.05 wt. %, Aldrich, Milwaukee, WI, USA) were used to determine the cation exchange capacity (CIC). Toluene (C_6_H_5_CH_3_, 99.8 wt %, Merck, Darmstadt, Germany), acetone (CH_3_COCH_3_, ≥99.5 wt. %, Merck, Darmstadt, Germany), absolute ethanol (CH_3_CH_2_OH, ≥99.5 wt. %, Merck, Darmstadt, Germany) and deionized water were used as solvents.

### 4.2. Organoclays Based on Bentonite and Kaolinite Synthesis

Syntheses of organoclays based on bentonite and kaolinite were carried out through the covalent anchorage of tClVS on their surface. This procedure was previously published [13]. For this, 10.0 g of clay in toluene were added and subjected to ultrasound (M3800-E, 40 KHz, 130 watts, Branson Ultrasonics™, Danbury, CT, USA) for 1 h for its adequate dispersion. Next, dispersion was heated to 95 °C in a reflux unit and 1.0 g of tClVS (10% *w*/*w* with respect to the amount of clay used) was added and kept in continuous reflux for 24 h at 95 °C. Later, organoclay was filtered, washed with acetone, and dried at 60 °C for 24 h. This procedure was carried out for both bentonite and kaolinite, obtaining the bentonite–tClVS and kaolinite–tClVS organoclays, respectively.

### 4.3. Geomimetic Soil Conditioners Synthesis

Synthesis of geomimetic composites was carried out through the polymerization via free radicals of the NaPSS in the presence of organoclays (i.e., bentonite–ClVS and kaolinite–tClVS). For the above, 5.0 g of organo-clay was taken and dispersed in 200.0 mL of water under constant stirring at 1000 rpm for 4 h. Subsequently, the required amount of NaSS was added to prepare composites with 10.0 and 20.0 wt. % of organoclay. Next, ammonium persulfate was added at 1.0 mol % with respect to the amount of NaSS and left under constant stirring at 1000 rpm for 2 h. Mixture was heated continuous at 70 °C in at reflux equipment under stirring at 1000 rpm for 4 h. Composites were filtered, washed with distilled water, and dried in an oven at 105 °C for 24 h. Finally, the geomimetic composites were crushed and sieved through a 2.0 mm mesh. The composites obtained were identified as Kao-NaSS-10, Kao-NaPSS-20, Bent-NaPSS-10 and Bent-NaPSS-20. The composition and the identification of each material obtained are shown in Table 2.

### 4.4. Structural and Morphological Characterization

Structural characterization of organoclays was carried out by Fourier Transform Infrared Spectroscopy of Attenuated Total Reflectance (IR-ATR, IRAffinity-1, Shimadzu, Kyoto, Japan) and spectral analysis using Functionally Enhanced Derivative Spectroscopy (FEDS) [53]. Morphological characterization of organoclays was studied by scanning electron microscopy (SEM, Phenom Pro X, ThermoFisher Scientific, Waltham, MA, USA) at an acceleration voltage of 15 kV. For SEM observation, the particles were pasted on carbon tape as background and coated with gold using a sputtering technique. The analysis of its surface composition was performed by energy dispersive X-ray spectroscopy (EDS, Phenom Pro X, ThermoFisher Scientific, Waltham, MA, USA). Also, the dynamic light scattering technique (DLS, Zetasizer LAB, Malvern Panalytical, Malvern, UK) was used to study the size and charge of the organoclay particles. For DLS characterization, dispersions of the particulate material in water were prepared at concentrations of 0.1 mg/mL and measurements were performed at 25 °C. The Kao-NaPSS-10, Kao-NaPSS-20, Bent-NaPSS-10 and Bent-NaPSS-20 composites were characterized structurally by IR-ATR-FEDS, morphologically by SEM and their elemental composition was determined by EDS.

### 4.5. Thermal Characterization

Thermal properties of hybrid composites, based on kaolinite–NaPSS and bentonite–NaPSS, were studied by thermogravimetric analysis (TGA, TA Q50, TA Instruments, New Castle, DE, USA), using an inert nitrogen atmosphere in a temperature range from 40 to 1000 °C and a heating ramp of 15 °C/min. For the analysis by differential scanning calorimetry (DSC, DSC25, TA Instruments, New Castle, DE, USA), the material thermal memory was eliminated by heating from room temperature at 300 °C with a ramp of 15 °C/min. Next, isothermal heating at 300 °C for three minutes, and a 25 °C/min cooling ramp from 300 °C to −30 °C. Finally, Differential scanning calorimetry (DSC) analysis was performed by heating from −30 °C to 300 °C with a ramp of 15 °C/min.

### 4.6. Water Sorption Capacity Study

*WSC* evaluation of clays, organoclays, and geomimetic composites was performed by tea bag method [54]. For this, approximately 0.2 g of material (*M*) was placed in a tea bag (*Tb*) and sealed. Subsequently, the *M* + *Tb*, (*MTb*) was immersed in deionized water for 24 h. Finally, the sample was removed from the water and *WSC* was determined gravimetrically by the following equation:

(1) $$WSC\;\left(\%\right)=\frac{\left({MTb}_{24}-{Tb}_{24}-M\right)}{M}*100$$

where *MTb*_24_ is the weight of *MTb* after 24 h submerged in water; *Tb*_24_ is the blank, that is, the sorption of water by the tea bag after 24 h submersion. Tests were carried out in triplicate and a blank experiment was carried out to determine the amount of water absorbed by the tea bag.

### 4.7. Cation Exchange Capacity Study

Cation exchange capacity (CEC) of clays, organoclays and geomimetic composites were determined using 1.0 N ammonium acetate method defined by the Agustín Codazzi Geographic Institute [55]. For this, a defined quantity of the material (1.0 g) is placed in contact with 10.0 mL of 1.0 N ammonium acetate solution to promote cation exchange reactions. Subsequently, sample was filtered, and ammonium remaining in materials was exchanged with sodium ions. Finally, exchanged ammonium was quantified by formaldehyde titration with NaOH solution. The endpoint determination of the reaction was carried out using phenolphthalein in a 1% ethanolic solution as an indicator. The test was performed in triplicate for each sample and a control blank was included for the experiment.

## Figures and Tables

**Table 1:** Elemental contents determined by EDS, size, zeta potential, WSC and CEC of clays (kaolinite and bentonite) and modified clays (kaolinite–tClVS and bentonite–tClVS).

Sample	Elemental Composition (% *w*/*w*)						Particle Size (nm)	Ζeta Potential (mV)	WSC (% *w*/*w*)	CEC (meq/100 g)
	O	Si	Al	Fe	C	Cl				
Bentonite	---	---	---	---	---	---	268 ± 16	−38.3 ± 0.2	621 ± 19	104 ± 3
Kaolinite	---	---	---	---	---	---	555 ± 20	−38.8 ± 1.3	210 ± 90	57 ± 4
Bentonite–tClVS	31.55	31.75	13.02	11.90	7.72	2.46	1144 ± 333	−33.3 ± 4.7	300 ± 38	43 ± 5
Kaolinite–tClVS	52.07	19.96	7.09	6.49	8.65	5.74	776 ± 34	−16.6 ± 2.3	145 ± 43	38 ± 2

O: oxygen, Si: silicon, Al: aluminum, Fe: iron, C: carbon and Cl: chlorine. The elemental composition was determined by EDS, the particle size and Z potential were determined by DLS.

**Table 2:** Sample identification, composition and elemental analysis determined by EDS of PCNCs based on kaolinite–NaPSS and bentonite–NaPSS.

Composition (% *w*/*w*)	Sample			
	Kao-NaPSS-10	Kao-NaPSS-20	Bent-NaPSS-10	Bent-NaPSS-20
NaSS	90.0	80.0	90.0	80.0
kaolinite–tClVS	10.0	20.0	---	---
bentonite–tClVS	---	---	10.0	20.0
O	44.91	34.59	46.35	41.17
C	18.75	35.97	13.50	16.07
S	8.60	13.74	10.08	13.27
Na	17.54	11.14	12.10	11.44
Si	0.91	1.96	5.58	8.56
Al	1.43	2.59	3.32	4.40

O: oxygen, C: carbon, S: sulfur, Na: sodium, Si: silicon and Al: aluminum. The elemental composition was determined by EDS.

**Table 3:** TGA and DSC results of PCNCs based on bentonite–NaPSS and kaolinite–NaPSS.

Parameter		Sample			
		Kao-NaPSS-10	Kao-NaPSS-20	Bent-NaPSS-10	Bent-NaPSS-20
Stage 1	Initial T (°C)	40	40	40	40
	Final T (°C)	340	350	340	335
	Δm (%)	15.92	16.00	15.77	14.57
Stage 1I	Initial T (°C)	340	350	340	335
	Final T (°C)	600	600	630	630
	Δm (%)	24.01	26.06	28.09	25.88
Stage 1II	Initial T (°C)	600	600	630	630
	Final T (°C)	1000	100	1000	1000
	Δm (%)	30.98	20.76	15.24	13.98
Residual mass	(%)	29.09	37.18	40.90	45.57
Tg	(°C)	112.1	113.4	109.1	118.6

Tg: glass transition temperature.

## Data Availability

The data presented in this study are openly available in article.
